# Evaluation of the Antimalarial Activity of Crude Extract of *Eucalyptus globulus* Labill. Leaf against *Plasmodium berghei* in Mice

**DOI:** 10.1155/2021/7068999

**Published:** 2021-09-20

**Authors:** Mihret Ayalew, Azmeraw Bekele

**Affiliations:** ^1^Department of Pharmacology, Institute of Health, Jimma University, Jimma, Ethiopia; ^2^Department of Social and Administrative Pharmacy, Institute of Health, Jimma University, Jimma, Ethiopia

## Abstract

**Introduction:**

Traditional medicinal plants are used as a common source of developing new and effective antimalarial drugs. *E. globulus* leaf has been used in the traditional management of malaria in different countries, including Ethiopia. However, there is no *in vivo* study done on the antimalarial activity of the plant. Thus, this study aimed to evaluate the antimalarial activity of crude extract of *E. globulus* Labill. leaf in *P. berghei*-infected mice.

**Method:**

The fresh leaves of *E. globulus* were collected, washed, air-dried, and made as coarse powder. Either sex of mice aged 6 to 8 weeks was used in the experiment. The antimalarial activity of the crude extract was tested in four-day suppressive, curative (Rane's), and prophylactic (repository) tests. The parameters like level of parasitemia, packed cell volume, body weight, rectal temperature, and mean survival time were recorded. The analysis of the data was done with SPSS version 20 with a 95% confidence interval in one-way ANOVA followed by Tukey's post hoc test.

**Results:**

In all three antimalarial test models, the extract of leaf of *E. globulus* at all three doses suppressed the level of parasitemia significantly (*p* < 0.001), increased survival time (*p* < 0.05 to *p* < 0.001), and prevented a decrease in body weight as compared to the negative control. The middle and large doses of the extract also decreased loss of body temperature (*p* < 0.05 to *p* < 0.001) compared to the negative control. Reduction of packed cell volume was prevented within the three test doses of the extract in both curative and prophylactic tests and middle and large doses in the 4-day suppressive test compared to the negative control.

**Conclusion:**

The crude extract of the plant showed promising antimalarial activity. This supports the traditional use and the *in vitro* test result of the plant.

## 1. Introduction

Even though there is a decline in the incidence of malaria, and associated morbidity and mortality as time goes by, malaria continues to be one of the world's major health problems. According to the 2019 World Malaria Report, there were 228 million cases of malaria, which shows a 3-million decrement compared to 2017, throughout the world. Nearly all malarial cases were recorded in the African region, which was accounting for 93%. The other malaria prevalent regions were Southeast Asia (3.4%) and Eastern Mediterranean (2.1%). Under-five years aged children were the most vulnerable group, which was accounting for 67% of the total deaths from malaria in 2018 [1].

In 2017, 60% of the Ethiopian population was identified as at risk of malaria contracting. From June 2016 to July 2017, there were 1,755,748 cases of malaria and 346 deaths. *P. falciparum* and *P. vivax* are major malaria-causing parasites in Ethiopia. Of 1,530,739 confirmed cases of malaria, 1,059,847 were caused by *P. falciparum* and 470,892 were caused by *P. vivax* [2].

Resistance to conventional antimalarial drugs has become a great challenge in the prevention, control, and eradication of malaria. Particularly, *P. falciparum* develops resistance to almost all antimalarials including artemisinin-based therapies [3–5]. *P. vivax* was also found to develop widespread resistance to chloroquine [6–8].

Since, the discovery of malaria, the use of traditional herbal remedies to fight malaria has become well known in malaria-endemic countries around the world. Numerous ethnobotanical, *in vitro,* animal, and clinical studies have been conducted concerning traditional herbal medicines used for malaria management throughout the world. The quinine and artemisinin derivatives are derived from traditional herbs [9, 10].

In Ethiopia, where the flora is diverse, it is common to use different parts of traditional medicinal plants in various preparations to combat malaria. Many plant species used for the management of malaria have been specified within ethnobotanical studies conducted in various regions of Ethiopia [11–14]. In addition, the results of various animal experiments on different types of traditional medicinal plants showed promising antimalarial activity [15–17].

*E. globulus* Labill., also called “nech-bahir zaf” (Amharic), is one of the traditional medicinal plants used to control malaria in Ethiopia. Drinking the boiling of the leaves of *E. globulus* is used to treat malaria in Hawassa city, southern Ethiopia [18]. In addition, a steam bath of the whole body with the boiling of the leaf of *E. globulus* is used to treat malaria by the Sidama people of Boricha District, Sidama Zone, South Region of Ethiopia [11].

The leaves of *E. globulus* are rich in eucalyptol (1,8-cineole), *α*-pinene, and (−)-globulol. Eucalyptol, the major phytochemical component, is responsible for several pharmacological activities [19–21]. Although age variation affects the amount of constituent of alpha-pinene and eucalyptol, all are found in all age leaves of *E. globulus* [22].

Various reported pharmacological activities of *E. globulus* include analgesic and anti-inflammatory activity [23], antibacterial activity [24], anthelmintic activity [25], antioxidant [13, 21], antipyretic [26], and lipid peroxidation inhibitory activity [27], and insecticidal activity [28]. In addition, *E. globulus* leaf extract reported having *in vitro* antiplasmodial activity against chloroquine-sensitive and resistance *P. falciparum* with IC_50_ of 16.80 ± 8.35 *µ*g/ml and 26.45 ± 3.32 *µ*g/ml, respectively [29]. However, *in vivo* studies to test the antimalarial activity of plant have not yet been conducted. Thus, this study aims to evaluate the antimalarial activity of the plant in mice.

## 2. Materials and Methods

### 2.1. Plant Material

The fresh leaves of *E. globulus* were collected from Azezo, Central Gondar, Gondar, Ethiopia, on 12 December 2020. The identification and authentication of the leaves were done by the botanist at the University of Gondar, Gondar, Ethiopia, and registered with voucher No. 001MAT/2020. The leaves were washed with clean water to remove dusty particles. Then, the leaves were air-dried under shade and made as coarse powder with clean mortar and pestle.

### 2.2. Extraction of the Plant Material

The resulting leaf powder was measured on a digital balance (EPH400 Abron Exports) and weighed 750 g. This powder was subjected to cold maceration with 80% methanol in powder to solvent ratio of 1 : 6 in Erlenmeyer flask for 72 hours at room temperature. After shaking the extract occasionally, the filtrate was separated from marc using gauze followed by Whatman Filter paper No. 1 after 72 hours. The marc was remacerated twice in the same volume of the solvent. Later on, the macerates were combined and dried at 40°C by hot air oven (Medit Medizintechnik, Germany). The extract was further dried by Lyophilizer (LabFreez Instruments, Germany). Finally, the dried extract was transferred into a well-covered glass and put in the refrigerator at 4°C until use.

### 2.3. Animals

Swiss albino mice 6 to 8 weeks of age were used in the test. The mice were maintained in a room at 12 hours light and dark cycle and fed on a standard pellet diet and water *ad libitum.* The mice were acclimatized for a week in a laboratory condition before the experiment. Mice were handled and used according to international guidelines for the animals used in the experiments [30].

### 2.4. Acute Oral Toxicity Testing

The acute oral toxicity of 80% methanol extract of leaf of *E. globulus* was tested according to Organization for Economic Cooperation and Development (OECD) guideline 425 [31]. The acute toxicity of leaf extracts was determined using 5 healthy female mice. The mice were fasted for 4 hours before and 2 hours after the test for food, but not water. The initial dose was determined by performing a sighting study containing administration of 2000 mg/kg of extract via oral gavage for one mouse. However, no death was recorded during the first 24 hours, and the remaining 4 mice were treated with the same dose of the extract. The mice were monitored every 30 minutes for the first 4 hours and every day for two weeks for common symptoms of toxicity.

### 2.5. Parasite Inoculation

The parasite was maintained by serial passage of blood from donor mice to noninfected ones. An infected mouse with a parasitemia level of 30–37% was used as a donor [17]. The donor mice were killed by cervical dislocation, and blood was collected from the jugular vein into a heparinized tube. Depending on the parasitemia level of the donor mice and the red blood cell count of the normal mice, the collected blood was diluted with normal saline (0.9%) to obtain the standardized parasitic inoculum with 10^7^ pRBCs in 0.2 ml of suspension. Then, the healthy mice were inoculated intraperitoneally (IP) with the standardized inoculum.

### 2.6. Grouping and Dosing of Animals

The mice were randomly divided into five groups each with six. Group one (GI) was assigned as a negative control that was received 10 ml/kg of distilled water, whereas group two (GII) was leveled as a positive control that was treated with 25 mg/kg of chloroquine. The remaining groups (GIII, GIV, and GV) were treated with 100 mg/kg, 200 mg/kg, and 400 mg/kg of the extract, respectively. These doses of the extract were determined based on evidence from a previous study that stated 1/10^th^ of 2000 mg/kg corresponds to 200 mg/kg, whereas half and double of 200 mg/kg correspond to 100 mg/kg and 400 mg/kg, respectively [32].

### 2.7. Determination of Antimalarial Activity

#### 2.7.1. Four-Day Suppressive Test

This test was conducted according to the method used in Peters 4-day suppressive test [33]. In total, 30 mice were infected with the standard pRBC inoculum on the first day (D0). Two hours after infection, mice were grouped and treated as described in the grouping and dosage section. Treatment was continued with once-daily administration until the 5^th^ day (D4). On the 5^th^ day, blood was collected from the tail vein of each mouse to determine parasite levels. The mice's body weight, rectal temperature, and packed cell volume (PCV) were also recorded just before infection (D0) and after treatment (D4). From the first day of the experiment, the mice were followed for 28 days to determine the mean survival time (MST) of the mice for each group. The percent change (% change) of body weight, rectal temperature, and PCV was determined as follows:

(1) $$\begin{array}{c} \%\text{ change of body weight}=\frac{\text{mean of body weight at}\left(D4-D0\right)}{\text{mean of body weight at }D0}\times 100,\\ \%\text{ change of rectal temperature}=\frac{\text{mean of rectal temperature at}\left(D4-D0\right)}{\text{mean of rectal temperature at }D0}\times 100,\\ \%\text{ change of PCV}=\frac{\text{mean of PCV at}\left(D4-D0\right)}{\text{mean of PCV at }D0}. \end{array}$$

#### 2.7.2. Curative (Rane's) Test

The method used by Ryley and Peters [34] was applied to evaluate the effect of *E. globulus* leaf extract against already established infection. On the first day (D0), 30 mice were infected with the standard inoculum of pRBCs and left untreated till the 4^th^ day (D3). On the fourth day (D3), the mice were grouped and treated as described earlier. The treatment was continued as a daily dose until the 7^th^ day (D6). The parasitemia level that was monitored began from the day of treatment initiated (D3) to the 8^th^ day (D7). Other parameters including body weight, rectal temperature, and PCV were also measured on the day just before the treatment (D3) and the D7. The mice were followed for 28 days to determine the MST. The percent change (% change) of body weight, rectal temperature, and PCV was determined as follows:

(2) $$\begin{array}{c} \%\text{ change of body weight}=\frac{\text{mean of body weight at}\left(D7-D3\right)}{\text{mean of body weight at }D3}\times 100,\\ \%\text{ change of rectal temperature}=\frac{\text{mean of rectal temperature at}\left(D7-D3\right)}{\text{mean of rectal temperature at }D3}\times 100,\\ \%\text{ change of PCV}=\frac{\text{mean of PCV at}\left(D7-D3\right)}{\text{mean of PCV at }D3}\times 100. \end{array}$$

#### 2.7.3. Prophylactic (Repository Test)

The prophylactic activity of *E. globulus* leaf extract was tested based on the method applied by Peters [35]. On the first day (D0) of the test, 30 mice were randomly grouped and treated as mentioned earlier. The treated mice were left uninfected until the 5^th^ day (D4). On D4, the mice were infected with the standard inoculum of pRBCs intraperitoneally. On the 8^th^ day (D7), 72 hours after infection, the level of parasitemia was determined by taking blood from the tail vein of the mice. The PCV, body weight, and rectal temperature were also determined on the day just before the treatment (D0) and at the end of the treatment (D7). The MST of the mice in a group was determined by following up for 28 days. The percent change (% change) of body weight, rectal temperature, and PCV was determined as follows:

(3) $$\begin{array}{c} \%\text{ change of body weight}=\frac{\text{mean of body weight at}\left(D7-D0\right)}{\text{mean of body weight at }D0}\times 100,\\ \%\text{ change of rectal temperature}=\frac{\text{mean of rectal temperature at}\left(D7-D0\right)}{\text{mean of rectal temperature at }D0}\times 100,\\ \%\text{ change of PCV}=\frac{\text{mean of PCV at}\left(D7-D0\right)}{\text{mean of PCV at }D0}\times 100. \end{array}$$

#### 2.7.4. Determination of Parasitemia and Mean Survival Time

Parasitemia level in each test was determined by taking blood from the tail vein of the mice. The blood was applied on the microscope slide to make a thin smear of blood followed by fixation using absolute methanol and stained with 10% Giemsa stain for 15 minutes. Then, the slides were washed with tap water and dried at room temperature. Finally, the pRBCs were counted under a light microscope with a magnification power of 100x. At least, three fields per slide were counted to determine the level of parasitemia. The percentage parasitemia and parasitemia suppression were calculated using the following formulas [36]:

(4) $$\begin{array}{c} \text{\% parasitemia}=\frac{\text{total number of pRBCs}}{\text{total number of RBCs}}\times 100\text{,}\\ \text{\% parasitemia suppression}=\frac{\text{\%parasitemia in the}\left(\text{control group}-\text{treated group}\right)}{\text{\% parasitemia in the control group}}\times 100\text{.} \end{array}$$

The MST was determined by using the following formula [37]:

(5) $$\begin{array}{c} \text{MST}=\frac{\text{total number of days mice survived}}{\text{total number of mice}}\times 100. \end{array}$$

#### 2.7.5. Determination of Packed Cell Volume

Blood from the tail vein of each mouse was taken into the heparinized capillary tube filled to 3/4^th^ of its height and sealed well at the opening end. The sealed tubes were placed in a microhematocrit centrifuge by making the sealed end upward. Then, the blood was centrifugated at 12000 rpm for 15 minutes. After all, the tubes were taken out of the centrifuge, and the PCV was determined using a Micro-Hematocrit Reader. The PCV was determined using the following formula [38]:

(6) $$\begin{array}{c} \text{PCV}=\frac{\text{volume of erythrocytes in a given volume of blood}}{\text{total blood volume}}\times 100. \end{array}$$

### 2.8. Data Analysis

The result of the study was expressed as mean ± standard error of the mean (SEM). One-way analysis of variance (ANOVA) followed by post hoc Tukey's test within SPSS version 20 at 95% confidence interval was used.

## 3. Results

### 3.1. Acute Oral Toxicity Test

Physical and behavioral observations of experimental mice for any signs of toxicity including behavioral changes in movement activity, feeding, water intake, grooming, lethargy, and other signs of weakness or distress revealed no signs of toxicity and death of mice during 24 hours of dosing and for 2 weeks thereafter. This refers to the fact that LD_50_ value of the 80% methanol extract of *E. globulus* exceeded 2000 mg/kg in mice.

### 3.2. Four-Day Suppressive Test

This test result showed that *E. globulus* leaf extract at three doses significantly inhibited parasite levels compared with the control group (*p* < 0.001). In contrast, the standard drug (chloroquine 25 mg/kg) significantly inhibited blood parasite levels (*p* < 0.001) compared with 3 doses of the extract. At doses of 100, 200, and 400 mg/kg extract, the parasitemia inhibition rates were 50.00%, 60.81%, and 63.41%, respectively (Table 1). The mean survival time of mice was also significantly increased at all tested doses of the extract compared with controls (*p* < 0.01 to *p* < 0.001).

Parasite-induced weight loss was significantly reduced compared with controls (*p* < 0.05, 100 mg/kg and *p* < 0.001, 200 and 400 mg/kg). In addition, as shown in Table 2, the reduction in rectal temperature was significantly reduced when a middle and large dose of extract was used. The standard drug significantly reduced both weight loss and rectal temperature loss compared to three doses of extract and negative control (*p* < 0.01 to *p* < 0.001).

The PCV measurements showed that the extracts at 200 mg/kg and 400 mg/kg significantly prevented the decrease in PCV compared to the negative control (*p* < 0.001). In addition, the extract at 400 mg/kg had a comparable effect to the standard drug in preventing the decrease in PCV (Table 3).

### 3.3. Rane's Test

Results from a study of established infections showed significant inhibition of parasite blood levels at all three doses of the extract compared to controls (*p* < 0.001). However, at three doses of the extract, the degree of inhibition was significantly lower than that of standard drugs (*p* < 0.001, 100 and 200 mg/kg and *p* < 0.01, 400 mg/kg). Mean survival time in mice was also increased (*p* < 0.05 to *p* < 0.001) at all test doses of extract compared to controls (Table 4). The weight loss effect of infection was significantly reduced in the extract (*p* < 0.01, 100 and 200 mg/kg and *p* < 0.001, 400 mg/kg) compared to negative controls. In addition, extracts at 200 mg/kg and 400 mg/kg significantly prevented the temperature drop compared to control (*p* < 0.001). The 400 mg/kg extract was as effective as standard drugs in preventing temperature loss (Table 5). Measurement of antihemolytic activity showed that the extract significantly reduced PCV reduction compared with the control at all doses (*p* < 0.001). The 400 mg/kg of the extract was as effective as the standard drug in preventing the reduction of PCV (Table 6).

### 3.4. Prophylactic/Repository Test

The results showed that all tested extract doses had a significant reducing effect (*p* < 0.001) on the degree of parasitemia compared to the control group. Mice survival was increased at all tested doses of the extract compared to controls (*p* < 0.05 to *p* < 0.001). All extract doses tested suppressed parasite blood levels and prolonged survival in mice, but the effect was significantly lower compared to the standard drug (*p* < 0.001) (Table 7). Rectal temperature and weight loss due to infection were significantly reduced with 200 mg/kg and 400 mg/kg extracts compared to negative controls (*p* < 0.001). However, all tested doses of the extract showed low activity compared to the prophylactic effect of standard drugs on rectal temperature and weight loss (Table 8). All three tested doses of the extract showed significant activity (*p* < 0.01 to *p* < 0.001) in reducing PCV. The 400 mg/kg crude extract had a comparable effect as the standard drug (Table 9).

## 4. Discussion

This study examined the acute oral toxicity and antimalarial activity of 80% methanol (hydromethanol) of the leaf of *E. globulus* extract. No signs of toxicity or animal death were observed. This shows that the plant can be safely used. The antimalarial activity of *E. globulus* extract was tested in three models including the 4-day suppressive test, the Rane's test, and the repository test.

The 4-day suppressive test is the most widely used antimalarial model and includes a comparison of blood parasite levels and mean survival time between treatment and no treatment to determine the antimalarial efficacy of compounds. *P. berghei* is a rodent malaria protozoan commonly used for the early identification of antimalarial compounds [39]. Compounds that reduced blood levels of parasites by more than 30% compared to negative controls were found to be active [40]. In line with this, three study doses of the extract showed greater than 30% blood parasite level suppression in all three antimalarial models.

Mice infected with *P. berghei* experienced a drop in body weight, body temperature, and destruction of red blood cells. Destruction of RBCs or hemolysis resulted in a decrease in mouse PCV and survival time. Plant extracts with malaria activity prevented reductions in body weight, body temperature, and hematologic abnormalities and were able to prolong the survival of mice [15, 17]. Consistent with this, *E. globulus* extract decreased loss of body weight, body temperature, PCV, and prolonging survival time in mice compared to the negative control.

Oxidative stress, which increases the release of reactive oxygen species and free radicals, is closely linked to malaria infection. It is associated with hemolysis (reduced PCV) and anemia [37]. Therefore, the improvement in the levels of PCV observed in the extracts can be attributed to the antioxidant activity of the study plant [13, 21] and the activity of membrane stabilization [27]. Alternatively, the extract's ability to decrease PCV reduction may be related to the prevention of parasite invasion and/or alter blood cell proliferation and increase red blood cell production [15].

Antimalarial activity of medicinal plants relies on their secondary metabolites including but not excluding flavonoids, alkaloids, terpenoids, and phenols. Altering the influx of essential nutrients to the parasite, direct cytotoxicity to the parasite, boosting host immunity, and inhibiting heme polymerization is the proposed antimalaria mode of action of secondary metabolites [37, 38]. Aqueous extract of leaf of *E. globulus* contains saponins, tannins, phenols, and glycosides [41]. Thus, the observed antimalarial activity of the extract may be due to the presence of such secondary metabolites.

Malaria infection activates the host's immune system and induces an inflammatory response. Common inflammatory mediators, such as prostaglandins and cytokines (tumor necrosis factor, interferon *γ*, and interleukin-1*β*), enhance the expression of intracellular adhesion molecules involved in the binding of parasitized RBCs parasites in the vascular endothelium [42]. In this regard, the anti-inflammatory effects of plants [23] may alter the pathogenesis of the parasite.

## 5. Conclusion

The hydromethanol extract of *E. globulus* exhibits relevant antimalarial activity, supporting the traditional use of the plant and *in vitro* study results. The observed antimalarial activity of the extract could be attributed to the presence of secondary malaria metabolites, antioxidant, and anti-inflammatory activity of the plant. This finding could be the starting point for future studies on the antimalarial activity of different solvent fractions and the isolation of active metabolite/s of the extracts.

## Figures and Tables

**Table 1:** Chemosuppressive activity of 80% the methanol leaf extract of *E. globulus* against *P. berghei* infection in mice.

Treatment	% parasitemia	% parasitemia suppression	Mean survival time (day)
DW	29.60 ± 2.55	00.00	6.00 ± 0.58
CE (mg/kg)			
100	14.80 ± 1.15	50.00^a3b3^	10.50 ± 1.18^a2b3^
200	11.60 ± 1.51	60.81^a3b3^	11.83 ± 1.01^a3b3^
400	10.83 ± 0.87	63.41^a3b3^	13.33 ± 0.88^a3b3^
CQ25 mg/kg	0.00 ± 0.00	100.00^a3^	28.00 ± 0.00^a3^

Data are expressed as mean ± SEM; *n* = 6. DW = distilled water, CE = crude extract, CQ = chloroquine, *a* = compared to negative control, *b* = compared to a standard drug, 1 = *p* < 0.05, 2 = *p* < 0.01, and 3 = *p* < 0.001.

**Table 2:** Rectal temperature and bodyweight of infected mice treated with 80% methanol leaf extract of *E. globulus* in the 4-day suppressive test.

Groups	Body weight (g)			Rectal temperature (°C)		
	D0	D4	% change	D0	D4	% change
DW	26.92 ± 0.83	22.50 ± 1.13	−16.58	37.25 ± 0.21	34.33 ± 0.42	−7.84
CE (mg/kg)						
100	27.67 ± 1.21	24.83 ± 1.15	−10.27^a1b3^	36.75 ± 0.21	34.25 ± 0.21	−6.80^b3^
200	27.58 ± 1.46	26.08 ± 1.36	−5.42^a3b3^	36.67 ± 0.21	35.08 ± 0.35	−4.32^a2b3^
400	26.92 ± 0.79	27.00 ± 0.62	0.41^a3b2^	37.00 ± 0.29	35.67 ± 0.31	−4.96^a1b3^
CQ25 mg/kg	27.33 ± 0.63	29.67 ± 0.76	8.54^a3^	36.92 ± 0.30	37.25 ± 0.17	0.92^a3^

Data are expressed as mean ± SEM; *n* = 6. DW = distilled water, CE = crude extract, CQ = chloroquine, *D*0 = day 0, *D*4 = day 4, *a* = compared to negative control, *b* = compared to a standard drug, 1 = *p* < 0.05, 2 = *p* < 0.01, and 3 = *p* < 0.001.

**Table 3:** Packed cell volume of *P. berghei*-infected mice treated with 80% methanol leaf extract of *E. globulus* in the 4-day suppressive test.

Groups	Packed cell volume		% change
	D0	D4	
DW	53.10 ± 1.80	41.50 ± 2.43	−22.61 ± 2.97
CE (mg/kg)			
100	51.00 ± 1.86	43.33 ± 2.17	−15.23 ± 1.40^b3^
200	51.00 ± 1.61	45.83 ± 1.68	−10.19 ± 0.98^a3b2^
400	49.33 ± 1.82	47.00 ± 1.86	4.71 ± 1.75^a3^
CQ25 mg/kg	49.67 ± 1.69	49.83 ± 1.40	0.47 ± 1.21^a3^

Data are expressed as mean ± SEM; *n* = 6. DW = distilled water, CE = crude extract, CQ = chloroquine, *D*0 = day 0, *D*4 = day 4, *a* = compared to negative control, *b* = compared to a standard drug, 1 = *p* < 0.05, 2 = *p* < 0.01, and 3 = *p* < 0.001.

**Table 4:** Antimalarial activity of 80% methanol leaf extract of *E. globulus* against *P. berghei* in Rane's test.

Groups	% parasitemia (D7)	% parasitemia suppression	Mean survival time (days)
DW	33.30 ± 32.49	00.00	6.33 ± 0.61
CE (mg/kg)			
100	20.00 ± 2.24	40.00^a3b3^	9.17 ± 0.48^a1b3^
200	13.50 ± 1.54	59.50a3b3	11.33 ± 0.84^a3b3^
400	10.83 ± 1.33	67.50a3b2	13.83 ± 0.60a3b3
CQ 25 mg/kg	0.00 ± 0.00	100.00^a3^	28.00 ± 0.00^a3^

Data are expressed as mean ± SEM; *n* = 6. DW = distilled water, CE = crude extract, CQ = chloroquine, *D*7 = day 7, *a* = compared to negative control, *b* = compared to a standard drug, 1 = *p* < 0.05, 2 = *p* < 0.01, and 3 = *p* < 0.001.

**Table 5:** Rectal temperature and bodyweight of infected mice treated with 80% methanol leaf extract of *E. globulus* in Rane's test.

Groups	Weight (Gram)			Rectal temperature (°C)		
	D3	D7	% change	D3	D7	% change
DW	26.92 ± 0.83	22.8 ± 31.17	−15.37 ± 2.34	37.25 ± 0.21	34.50 ± 0.37	−7.39 ± 0.60
CE (mg/kg)						
100	27.67 ± 1.21	25.5 ± 81.00	−7.43 ± 0.57^a2b3^	36.75 ± 0.21	34.75 ± 0.17	−5.44 ± 0.48^b3^
200	27.58 ± 1.46	25.3 ± 31.43	−8.24 ± 0.47^a2b3^	36.67 ± 0.21	35.67 ± 0.31	−2.73 ± 0.50^a3b2^
400	26.92 ± 0.79	27.17 ± 0.69	0.99 ± 0.62^a3b2^	37.00 ± 0.29	36.42 ± 0.30	−1.58 ± 0.22^a3^
CQ25 mg/kg	27.33 ± 0.63	29.42 ± 0.82	7.59 ± 1.26^a3^	36.92 ± 0.30	36.92 ± 0.24	0.02 ± 0.61^a3^

Data are expressed as mean ± SEM; *n* = 6. DW = distilled water, CE = crude extract, CQ = chloroquine, *D*3 = day 3, *D*7 = day 7, *a* = compared to negative control, *b* = compared to a standard drug, 1 = *p* < 0.05, 2 = *p* < 0.01, and 3 = *p* < 0.001.

**Table 6:** Packed cell volume of *P. berghei*-infected mice treated with 80% methanol leaf extract of *E. globulus* in Rane's test.

Groups	Packed cell volume		
	D3	D7	% change
DW	52.5 ± 02.11	38.00 ± 2.25	−27.84 ± 1.93
CE (mg/kg)			
100	51.67 ± 2.26	46.00 ± 2.18	−11.02 ± 0.63^a3b3^
200	50.33 ± 2.12	47.33 ± 2.38	−6.12 ± 0.95^a3b2^
400	50.00 ± 1.91	49.17 ± 1.96	−1.67 ± 1.02^a3^
CQ25 mg/kg	49.83 ± 1.64	50.50 ± 1.28	1.48 ± 1.04^a3^

Data are expressed as mean ± SEM; *n* = 6. DW = distilled water, CE = crude extract, CQ = chloroquine, *D*3 = day 3, *D*7 = day 7, *a* = compared to negative control, *b* = compared to a standard drug, 1 = *p* < 0.05, 2 = *p* < 0.01, and 3 = *p* < 0.001.

**Table 7:** Chemoprophylactic activity of 80% methanol leaf extract of *E. globulus* against *P. berghei* in mice.

Groups	% parasitemia	% parasitemia suppression	Mean survival time (day)
DW	29.67 ± 1.17	0.00	6.17 ± 0.48
CE (mg/kg)			
100	16.50 ± 0.85	44.39^a3b3^	9.50 ± 0.99^a1b3^
200	15.83 ± 0.95	46.65^a3b3^	10.17 ± 0.87^a2b3^
400	14.00 ± 0.58	52.81^a3b3^	12.83 ± 0.65^a3b3^
CQ 25 mg/kg	5.67 ± 0.61	80.89^a3^	25.67 ± 0.42^a3^

Data are expressed as mean ± SEM; *n* = 6. DW = distilled water, CE = crude extract, CQ = chloroquine, *a* = compared to negative control, *b* = compared to a standard drug, 1 = *p* < 0.05, 2 = *p* < 0.01, and 3 = *p* < 0.001.

**Table 8:** Rectal temperature and bodyweight of *P. berghei*-infected mice treated with 80% methanol leaf extract of *E. globulus* in the prophylactic test.

Groups	Rectal temperature (°C)			Body weight (grams)		
	D0	D7	% change	D0	D7	% change
DW	37.25 ± 0.21	33.17 ± 0.61	−10.99 ± 1.17	27.50 ± 0.58	21.75 ± 0.89	−21.00 ± 2.23
CE (mg/kg)						
100	36.75 ± 0.21	33.67 ± 0.25	−8.38 ± 0.63^b3^	27.58 ± 0.76	23.83 ± 0.87	−13.60 ± 2.22^a1b3^
200	36.67 ± 0.21	34.58 ± 0.30	−5.69 ± 0.43^a3b3^	27.83 ± 1.05	25.75 ± 1.14	−7.56 ± 1.47^a3b2^
400	36.83 ± 0.21	35.42 ± 0.20	−3.84 ± 0.41^a3b2^	26.83 ± 0.74	25.67 ± 0.68	−4.32 ± 0.75^a3b1^
CQ 25 mg/kg	36.92 ± 0.30	37.00 ± 0.29	0.23 ± 0.54^a3^	27.00 ± 0.34	27.75 ± 0.28	2.81 ± 0.80^a3^

Data are expressed as mean ± SEM; *n* = 6. DW = distilled water, CE = crude extract, CQ = chloroquine, *D*0 = day 0, *D*7 = day 7, *a* = compared to negative control, *b* = compared to a standard drug, 1 = *p* < 0.05, 2 = *p* < 0.01, and 3 = *p* < 0.001.

**Table 9:** Packed cell volume of *P. berghei*-infected mice treated with 80% methanol leaf extract of *E. globulus* in the prophylactic test.

Groups	Packed cell volume		
	D0	D7	% change
DW	49.33 ± 1.26	40.67 ± 1.17	−17.67 ± 0.56
CE (mg/kg)			
100	50.33 ± 1.56	44.17 ± 1.40	−12.17 ± 0.48^a2b3^
200	49.50 ± 1.52	44.83 ± 1.82	−9.67 ± 0.42^a3b3^
400	50.33 ± 1.52	48.00 ± 1.57	−4.33 ± 0.21^a3^
CQ 25 mg/kg	50.00 ± 1.29	47.17 ± 1.56	−5.83 ± 0.48^a3^

Data are expressed as mean ± SEM; *n* = 6. DW = distilled water, CE = crude extract, CQ = chloroquine, *D*0 = day 0, *D*7 = day 7, *a* = compared to negative control, *b* = compared to a standard drug, 1 = *p* < 0.05, 2 = *p* < 0.01, and 3 = *p* < 0.001.

## Data Availability

All the necessary data have been incorporated in the article.

## Abbreviations

MST: Mean survival time
pRBCs: Parasitized red blood cells
PCV: Packed cell volume.
